# Population growth of phosphine resistant and susceptible populations of *Lasioderma serricorne* (F.) (Coleoptera:Anobiidae) exposed to different temperatures and commodities

**DOI:** 10.1007/s11356-023-26029-x

**Published:** 2023-02-28

**Authors:** Maria K. Sakka, Christos G. Athanassiou

**Affiliations:** Laboratory of Entomology and Agricultural Zoology, Department of Agriculture, Crop Production and Rural Environment, University of Thessaly, Phytokou Str, 38446 Nea Ionia, Magnesia Greece

**Keywords:** *Lasioderma serricorne*, Population growth, Phosphine resistance, Progeny production, Temperature, Commodity

## Abstract

The aim of this work was to investigate the population growth of *Lasioderma serricorne* (F.) with two populations with different susceptibility to phosphine (one resistant and one susceptible). Population growth was recorded on different days (35 days, 50 days, 65 days, 80 days, 95 days, and 110 days) in two different commodities: (a) mixed food consisted of wheat flour (10 parts) + cornmeal (10 parts) + brewers’ yeast (1.5 parts) and (b) wheat flour. Our results clearly indicate that both populations preferred mixed food compared to wheat flour for all combinations tested. Moreover, the increase in temperature from 25 to 30 °C showed a positive effect in some combinations in the population growth of both populations. In general, we found some differences in the production of offspring between the susceptible and the resistant population. Based on the results of the present study, population growth may provide critical information for the fitness advantages or disadvantages of each population.

## Introduction

During the last two decades, and especially after the withdrawal of methyl bromide and other active ingredients from their use in stored product protection (Fields and White 2002), phosphine gas has become the most commonly used insecticide for disinfestation of durable agricultural commodities (Afful et al. 2018; Nayak et al. 2020). In this context, a large number of studies underline the advantages of using phosphine in stored product protection as an easy-to-use, cost-effective, and residue-free method that can be applied in a wide range of commodities and facilities (Nayak and Collins 2008; Nayak et al. 2020). Moreover, phosphine has been found to be effective in the control of major stored product insect pest species, and at all life stages, with eggs being the most difficult life stage to control (Bell and Wilson 1995; Gourgouta et al. 2021). A series of published papers in commercial fumigations from many parts of the world illustrates that phosphine, if applied properly, can provide rapid disinfestation at intervals that usually range between 3 and 7 days (Aulicky et al. 2015; Chen et al. 2019; Agrafioti et al. 2020).

Despite the incontestable advantages of phosphine, the continuous use of this gas has led to the development of resistance of all major insect pest species of stored products (Collins et al. 2005; Holloway et al. 2016; Gautam et al. 2020). In a recent review, Nayak et al. (2020) provided a clear evidence that resistance to phosphine is currently a global phenomenon rather than a series of isolated incidences. For example, Opit et al. (2012) found that eight out of nine populations of the red flour beetle, *Tribolium castaneum* (Herbst) (Coleoptera: Tenebrionidae) sampled from storage facilities from Oklahoma, were found to be resistant to phosphine. Moreover, Agrafioti et al. (2019), in a series of samplings from Greece, found that from the eight populations of stored product beetles that had been sampled from different types of facilities, eight were recorded as resistant to phosphine. These works, along with many others from Australia (Cato et al. 2017; Afful et al. 2018), the USA (Gautam et al. 2020), China (Song et al. 2011; Huang et al. 2019), Morocco (Benhalima et al. 2004), Pakistan (Wakil et al. 2021), and elsewhere constitute essentially the need to investigate measures to mitigate this phenomenon, considering the importance of this gas for global agricultural trade and food security (Gautam et al. 2020; Sakka et al. 2020a; Sakka and Athanassiou 2021b).

It is generally expected that resistance to given insecticides is related to a specific fitness cost, particularly to life table characteristics of the resistant populations, such as longevity and fecundity (Saxena and Bhatia 1980; Fragoso et al. 2005). In the case of phosphine-resistant populations of *T. castaneum*, the lesser grain borer, *Rhyzopertha dominica* (F.) (Coleoptera: Bostryhidae) and the saw-toothed grain beetle, *Oryzaephilus surinamensis* (L.) (Coleoptera: Silvanidae), it has been reported a reduced progeny production capacity, as compared with the respective phosphine-susceptible populations. Moreover, Malekpour et al. (2016) have shown that phosphine-resistant populations of *T. castaneum* were less effective in locating food, which can be considered as a drawback in the dispersal capacities of these populations. Agrafioti et al. (2020) found that phosphine-resistant populations of *T. castaneum* and *Rhyzopertha dominica* (F.) (Coleoptera: Bostryhidae) were more slow-moving, as compared with the susceptible ones, so that these behavioral patterns could be utilized to quantify recovery after exposure to phosphine. However, there are studies that indicate that there are no differences in most of the above parameters among populations of *T. castaneum* with different susceptibility levels to phosphine (Sakka et al. 2020b). For instance, Fragoso et al. (2005) found that pyrethroid-resistant populations of the maize weevil, *Sitophilus zeamais* (Motsch.) (Coleoptera: Curculionidae), had similar fecundity patterns than susceptible ones, suggesting that there is no fitness cost of resistance toward this direction. Hence, it is likely that some of these differences that are noted between resistant and susceptible populations of insect pest species of stored products could be due to factors that may not be related to the occurrence of resistance, and their causes are much more complex than this single factor alone.

The cigarette beetle, *Lasioderma serricorne* (F.) (Coleoptera: Anobiidae), is a major threat to stored tobacco, causing serious losses (Ashworth 1993; Edde 2019). At the same time, this species has an extremely large variety of food preferences, ranging from amylaceous materials and dried fruit (Ashworth 1993) to weed species in the open field, such as thistles (Buchelos 1989). Mahroof and Phillips (2008) reported that the highest fecundity was observed in wheat flour and the lowest in tobacco for *L. serricorne*. Under different range temperatures and relative humidity, the development time ranged from 5 to 20 days for eggs, 18 to 101 days for larvae, 6 to 25 days for pupae, and 18 to 46 days for adults (Howe 1957). The optimum temperature for the development of *L. serricorne* is ranging from 29 to 35 °C and relative humidity from 65 to 75% (Edde 2019). Populations of *L. serricorne* have been found to be resistant to phosphine, with some of them to be classified as “strongly resistant” (Rajendran and Narasimhan 1994; Hori and Kasaishi 2005; Sağlam et al. 2015). Sağlam et al. (2015) reported that a single population of *L. serricorne* could survive exposure for 6 days at 600 ppm of phosphine, parameters that are usually lethal for most insect pest populations of stored products (Gourgouta et al. 2021; Sakka and Athanassiou 2021a). Moreover, Sakka and Athanassiou (2021a) quantified different populations of this species for their resistance to phosphine, indicating that resistance classification could be altered if different diagnostic protocols are used. Nevertheless, there are no data available so far for the population growth parameters of populations of *L. serricorne* with different susceptibility to phosphine. In this context, given that population growth is a key parameter that largely determines the rebound patterns after the termination of the fumigation, we used two populations of *L. serricorne* that had different susceptibility levels to phosphine, one susceptible and one resistant, to determine if there are differences in their population growth.

## Material and methods

### Insects

Τwo populations of *L. serricorne*, one susceptible and one resistant to phosphine, were used. These include the standard susceptible population which was reared in the Laboratory of Entomology and Agricultural Zoology (LEAZ) for decades and the field population which was taken from Malaysia in June 2016 (23–35 °C and 56% relative humidity (RH)) and was found resistant to phosphine (Sakka and Athanassiou 2021a). Both populations were cultured in wheat soft flour and were kept in incubator chambers set at 25 °C, 55% RH, and continuous darkness.

### Detection of phosphine resistance

The Food and Agriculture Organization (FAO) protocol, as described by FAO Plant Protection Bulletin (FAO 1975) and modified by Agrafioti et al. (2019), was used for the evaluation of the presence of phosphine resistance. In brief, twenty adults of each of the tested populations were placed in a 1.5 l glass jar and exposed to a phosphine concentration of 30 ppm for 20 h. After the termination of the exposure interval, active (i.e., capable of coordinated movement) and immobilized (i.e., not capable of coordinated movement) adults were recorded, as suggested by Athanassiou et al. (2019). The whole procedure was repeated three times, which were considered as replicates, with three sub-replicates each, with new phosphine production on each replicate (Sakka and Athanassiou 2021a). The phosphine production took place in a plastic canister by adding two tablets of a special formulation based on magnesium phosphide (same as the one used at the DDPTTK, Detia Degesch GmbH, Germany) and 50 ml of water. Phosphine concentrations within the jars were determined via quantitative gas chromatography (GC) using a Shimadzu GC-2010Plus (Shimadzu, Kyoto, Japan) instrument equipped with a GS-Q column (30 m long × 0.25 mm i.d., 0.25 μm film thickness, MEGA S.r.l., Italy) and a flame photometric detector set in the phosphorous mode. All gas samples from the jars were injected into the GC with a VICI 1-µl, gas-tight syringe (Sakka and Athanassiou 2021a).

### Population growth tests

The experiment was conducted in a phosphine-free environment in plastic vials (3 cm in diameter, 8 cm in high (Rotilabo Sample tins Snap on lid, Carl Roth, Germany). Two different commodities were used: (a) mixed food consisted of wheat flour (10 parts) + cornmeal (10 parts) + brewers’ yeast (1.5 parts) and (b) wheat flour. The commodities were untreated and uninfested and kept at ambient conditions until the beginning of the bioassays. Twenty grams of each commodity and twenty adults of each population were placed into each vial. The vials were placed in incubators set at two temperatures, 25 °C and 30 °C, 75% relative humidity (r.h.), and continuous darkness. The numbers of individuals produced were recorded after 35 days, 50 days, 65 days, 80 days, 95 days, and 110 days and were separated for individual life stages (larvae, pupae, and adults). Different series of vials were used for each time interval. The experiment was conducted with 3 replicates and 3 sub-replicates (= 9 vials for each combination).

### Data analysis

Prior to analysis, all data were tested for normalization and homogeneity using the O’Brien test or the Brown–Forsythe test. When variances were not equal, the data were transformed (*x*^2^, log (*x* + 1), log (*x*), or √*x*), while where the data were not normalized with any test of unequal variances, Wilcoxon tests were performed. The data were submitted separately for the number of larvae, pupae, and adults to two-way ANOVA with temperature and commodity as the main effects. Means were separated by the Tukey–Kramer HSD test at the 0.05 level. Untransformed means and standard errors are reported to simplify interpretation.

## Results

### Detection of phosphine resistance

Regarding immobilization after exposure to phosphine, all adults of the laboratory population were found to be immobilized after the 20-h exposure at 30 ppm (100% of immobilization), and for the resistant population, there was no immobilization.

### Population growth of *Lasioderma serricorne*

**Fig. 1:**
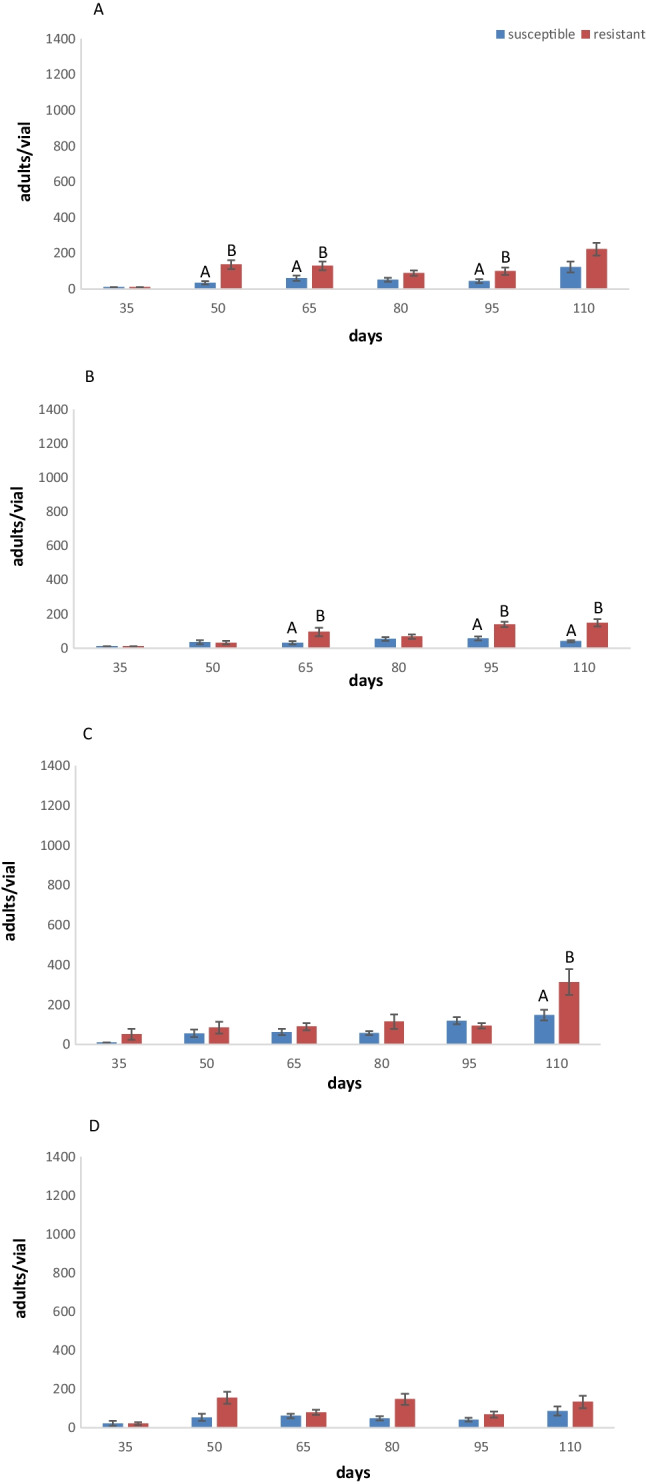
Mean number of adults per vial (± SE) produced by parental individuals of two populations of *Lasioderma serricorne* with different susceptibility to phosphine after 35 days, 50 days, 65 days, 80 days, 95 days, and 110 days on mixed food (flour + cornmeal + yeast) (**A**) and flour (**B**) at 25 °C and mixed food (**C**) and flour (**D**) at 30. Within each day and population, means followed by different letters indicate significant differences between populations; those with no letters exist, no significant differences were noted; in all cases, *df* = 1,17, HSD test at 0.05

The main effects as well as associated interactions were presented in Table 1. Regarding *L. serricorne* adults, at 25 °C, progeny production was higher for resistant as compared with susceptible on mixed food (Fig. 1A). On flour, low adult emergence was recorded for most of the days for both populations. The resistant population showed a higher number of adults after 110 days and the susceptible population after 80 days (Fig. 1B).

**Table 1:** ANOVA parameters and main effects and simple interactions for the number of adults, larvae, and pupae of two *Lasioderma serricorne* populations that were produced in two different diets and at two temperature levels (*error df* = *405*)

		Adults		*Larvae*		*Pupae*	
	*df*	*F*	*P*	*F*	*P*	*F*	*P*
Whole model	26	11.9	< 0.001	6.0	< 0.001	3.8	< 0.001
Intercept	1	680.9	< 0.001	167.6	< 0.001	79.0	< 0.001
Species	1	67.8	< 0.001	7.2	0.007	0.1	0.830
Temperature	1	6.8	0.009	1.9	0.171	2.2	0.140
Strain	1	15.9	< 0.001	11.4	< 0.001	6.6	0.010
Days	5	32	< 0.001	11.4	< 0.001	10.0	< 0.001
Strain × temperature	1	0.1	0.793	0.6	0.422	0.4	0.534
Strain × commodity	1	0.5	0.477	0.1	0.985	0.8	0.372
Strain × days	5	4.4	< 0.001	5.0	< 0.001	3.2	0.007
Temperature × commodity	1	0.1	0.945	1.9	0.167	5.2	0.023
Temperature × days	5	1.4	0.234	8.1	< 0.001	1.2	0.295
Commodity × days	5	6.2	< 0.001	2.1	0.059	2.4	0.035

At 30 °C on mixed food, low adult emergence was recorded for the susceptible population after almost all days (Fig. 1C). Moreover, the highest adult numbers were recorded after 50 days, 65 days, 80 days, and 110 days for the resistant population on mixed food (Fig. 1C). For both species, the higher number of adults was after 110 days. On flour, adult numbers were lower than on mixed food (Fig. 1D), but again, resistant populations had the highest adult numbers for all days (Fig. 1D). The susceptible population remains in very low adult emergence.

**Fig. 2:**
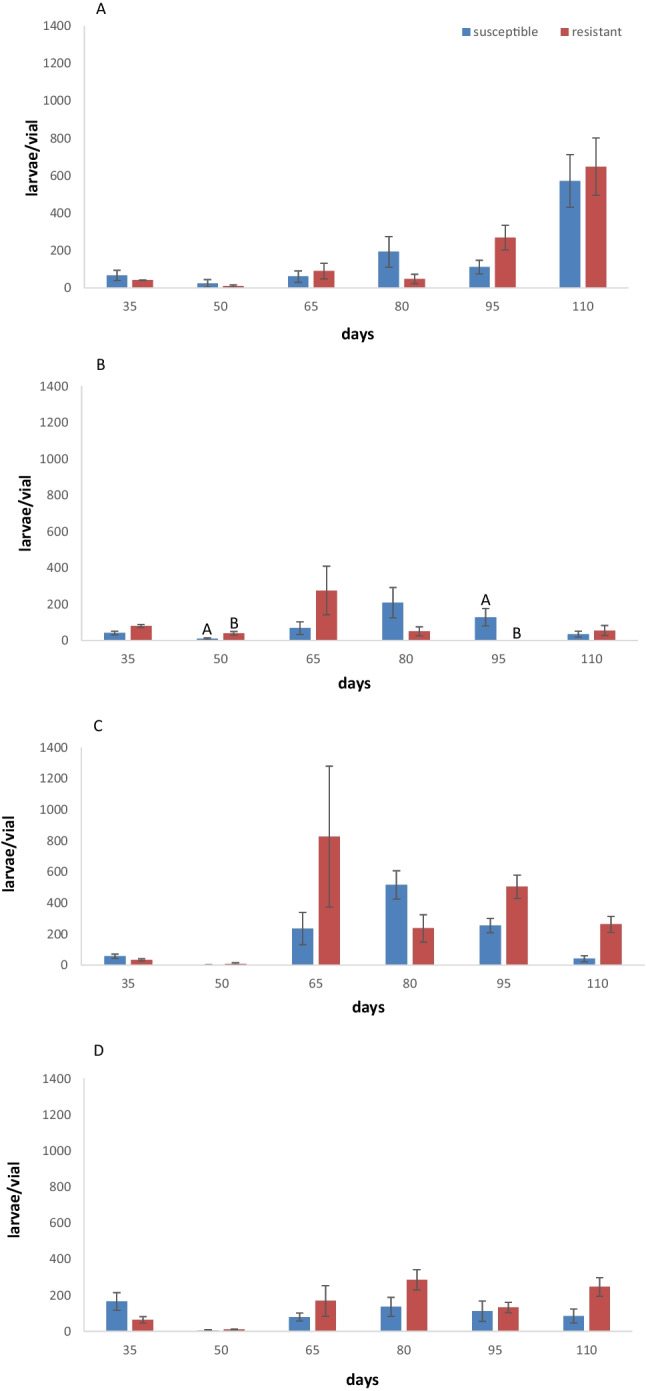
Mean number of larvae per vial (± SE) produced by parental individuals of two populations of *Lasioderma serricorne* with different susceptibility to phosphine after 35 days, 50 days, 65 days, 80 days, 95 days, and 110 days on mixed food (flour + cornmeal + yeast) (**A**) and flour (**B**) at 25 °C and mixed food (**C**) and flour (**D**) at 30 °C. Within each day and population, means followed by different letters indicate significant differences between populations; those with no letters exist, no significant differences were noted; in all cases, *df* = 1,17, HSD test at 0.05

At 25 °C, larval numbers were low after 35 days and 50 days for both populations on mixed food (Fig. 2A). High larval numbers were recorded for the resistant population after 95 days and 110 days and for the susceptible population after 35 days, 50 days, and 80 days on mixed food. On flour, the highest larval number was recorded for the resistant population after 65 days (Fig. 2B). Moreover, significant differences were noted between populations after 95 days (Fig. 2B).

At 30 °C, low larval numbers were recorded for both populations after 35 days and 50 days on mixed food. The higher larval numbers were recorded after 65 days for the resistant population (Fig. 2C). High larval numbers were recorded for the susceptible population after 80 days (Fig. 2C). On flour, larval numbers were low for both populations (Fig. 2D).

**Fig. 3:**
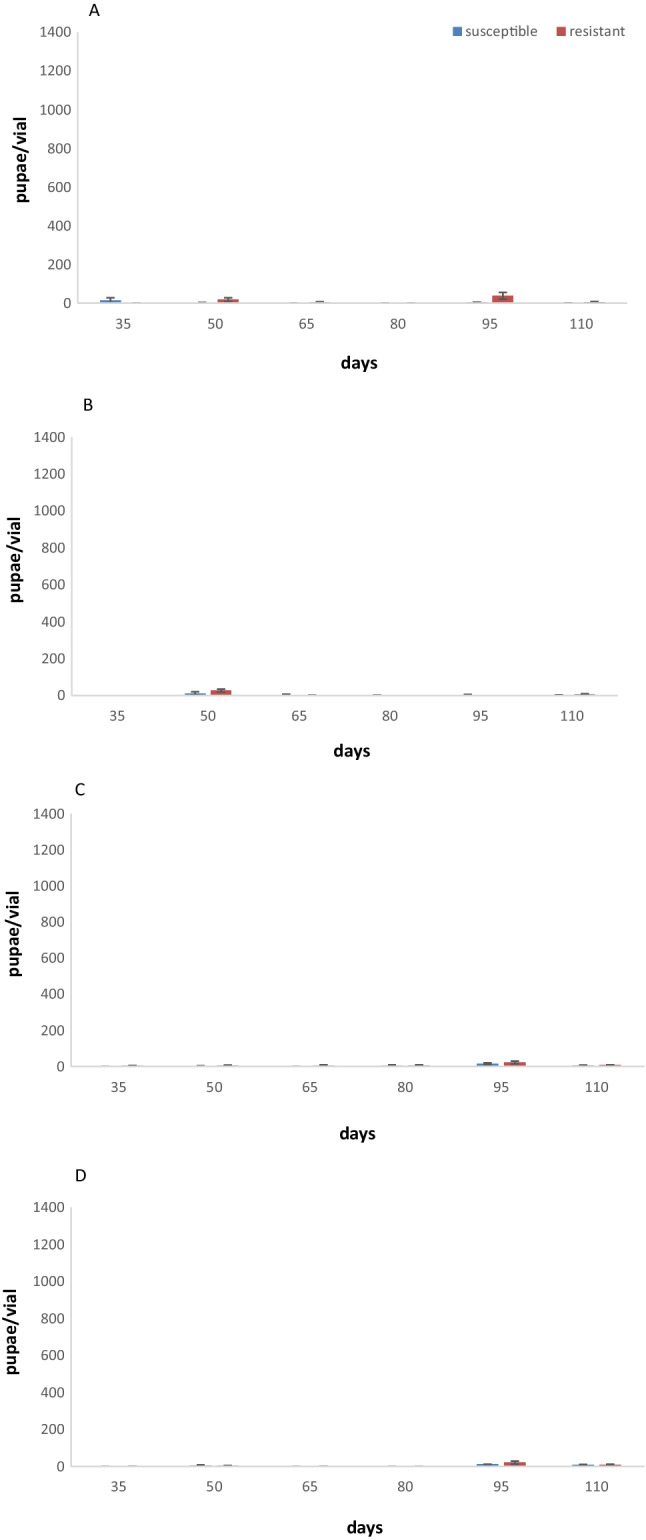
Mean number of pupae per vial (± SE) produced by parental individuals of two populations of *Lasioderma serricorne* with different susceptibility to phosphine after 35 days, 50, 65, 80, 95, and 110 days on mixed food (flour + cornmeal + yeast) (**A**) and flour (**B**) at 25 °C and mixed food (**C**) and flour (**D**) at 30 °C. Within each day and population, means followed by different letters indicate significant differences between populations; those with no letters exist, no significant differences were noted; in all cases, *df* = 1,17, HSD test at 0.05

At 25 °C, the number of pupal development is similar for both populations (Fig. 3A). On flour, there were no pupae for any of the populations after 35 days and 80 days, and very low numbers after 50 days, 65 days, 95 days, and 110 days (Fig. 3B).

At 30 °C for all experimental days, pupal numbers were higher for the resistant populations on mixed food (Fig. 3C). For the susceptible population, no or very low pupal numbers were recorded (Fig. 3C). On flour, the highest pupal numbers were recorded after 95 days (Fig. 3D).

## Discussion

The evaluation protocol that was used here as a diagnostic of phosphine resistance has been regarded as one of the most reliable for this purpose and has been used extensively in stored product insect populations, including *L. serricorne* (Sağlam et al. 2015; Sakka and Athanassiou 2021a). Still, the FAO protocol can be used as a first diagnostic to indicate tolerance or resistance, but cannot quantify resistance, i.e., separate strong from weak resistance in different insect populations, as this can be recorded through specialized bioassays or molecular markers (Champ and Dyte 1976; Chen et al. 2015). Hence, we are unaware if the resistant population tested here was strongly resistant or not, but the immobilization patterns that were recorded in our bioassays show that the adults of this population were not affected.

Although *L. serricorne* is a polyphagous species, the type of commodity plays a critical role in its development. Mahroof and Phillips (2008) reported that, among a series of substrates, this species can develop better in amylaceous materials, such as flour and less in tobacco. Still, the authors postulated that *L. serricorne* is dominant in tobacco due to the fact that this commodity is probably detrimental to most of the major stored product insects, and thus, *L. serricorne* has little competition during the infestation (Mahroof and Phillips 2008; Baliota et al. 2022). Our data show that the mixed diet was superior than the use of flour alone for all combinations tested. This was somehow expectable due to the presence of yeast that constitutes a rich protein for insect development. For instance, Kotsou et al. (2021) found that the increase in the percentage of yeast increased the population growth and the overall performance of the lesser mealworm, *Alphitobius diaperinus* (Panzer) (Coleoptera: Tenebrionidae), which is an authorized insect species for food and feed, and suggested that such an approach can be further utilized in mass rearing protocols.

**Fig. 4:**
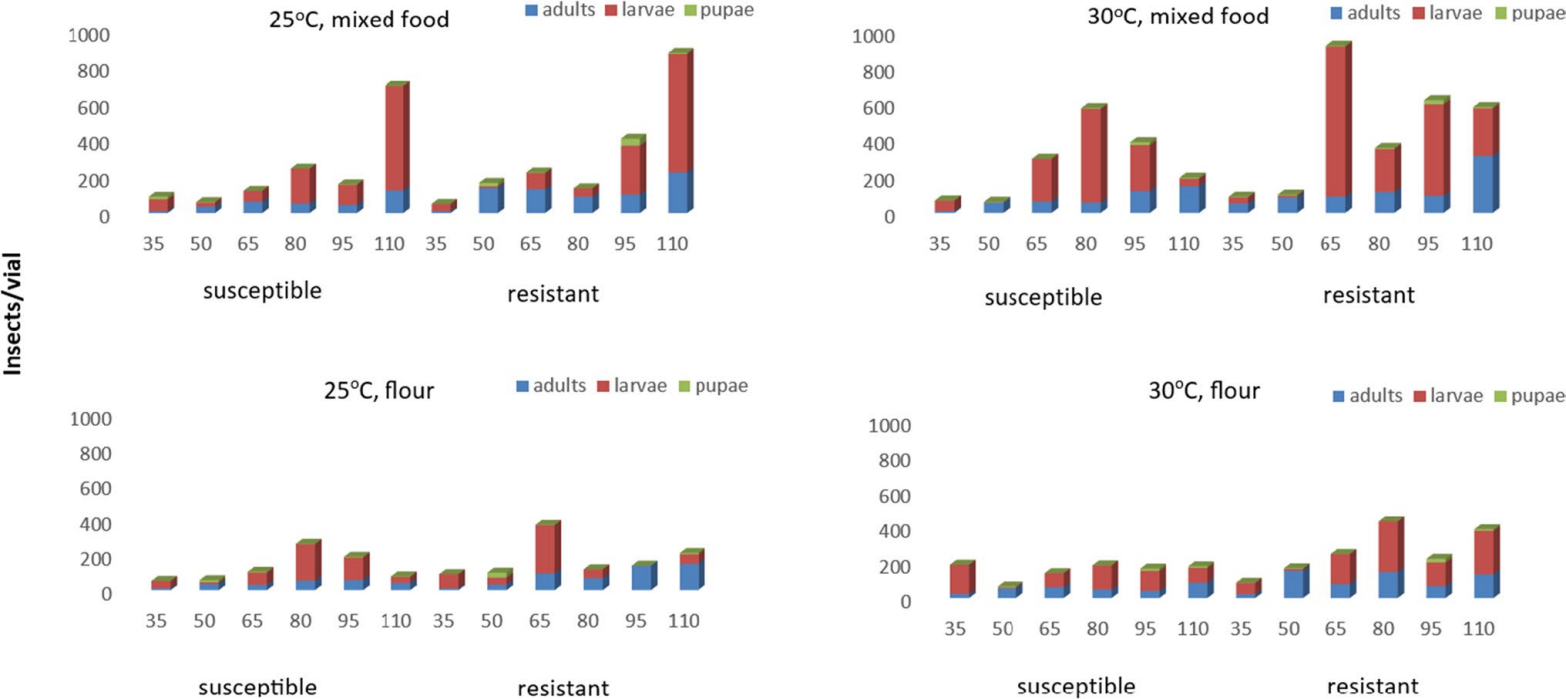
Overview of the *Lasioderma serricorne* results with all life stages for two populations (susceptible and resistant to phosphine)

Regarding the overall progeny production data, we found that the resistant population was able to produce more offspring than the susceptible one in most of our cases, a difference that was mostly evident at the final observation interval (110 days) (Fig. 4). Previously published papers provide dissimilar results regarding the progeny production capacity of phosphine-resistant insect populations, as compared with the susceptible ones, but most of the studies show that there are either no differences or the susceptible populations have higher population growth (Saxena and Bhatia 1980; Sousa et al. 2009). Thus, the highest population growth of the resistant *L. serricorne* population is unexpected, considering the previous data for other species. However, we assume that this might be partially related to the fact that the resistant population was recently sampled from the field, as opposed to the laboratory population that has been maintained at the laboratory for decades in flour, and hence, the latter may be more adapted to this commodity, while the field-collected individuals are likely to be more successful in food source exploitation. From a practical point of view, the fact that the resistant *L. serricorne* population was able to produce more progeny implies that this population can have more rapid colonization than the susceptible one and can cause considerable infestations in a short period of time. For *T. castaneum* adults, Athanassiou et al. (2019) found that phosphine-resistant individuals could recover rapidly after exposure to phosphine, a characteristic that theoretically allows them to distribute easily and colonize uninfected areas.

We also found that the superiority of the population growth of the resistant population over the respective figures of the susceptible one was evident only in some of the observation intervals. This is probably due to the changes in the ratios of the different life stages in the experimental vials, such as newly hatched larvae and especially eggs that remained undetected. In a recent study, Baliota et al. (2022) tested the development of *L. serricorne* to different types of tobacco and found that the different life stages of this species within the commodity were somehow analogous. Moreover, in that study, the authors have shown that the development of populations of *L. serricorne* with different susceptibility to phosphine was similar, regardless of the type of tobacco (Baliota et al. 2022). This suggests that, apart from the overall progeny production counts during the observation intervals, there were differences in the speed of development between the two populations tested.

Edde (2019) collected different studies on the biological parameters that determine the development of *L. serricorne* and considered 29–35 °C as the optimum temperature. The results of the present study show that the increase in temperature from 25 to 30 °C had a positive effect on the population growth of this species for both populations tested (Fig. 4). However, this was evident only in the case of the mixed diet, and only in some combinations. Baliota et al. (2022) found that the increase in temperature increased population growth in a similar way for both resistant and susceptible *L. serricorne* populations, but these data corresponded to tobacco and may not be directly comparable with the results of the current study. Nevertheless, as all vials contained the same amount of food, it is likely that progeny production could be limited by food exploitation parameters and insect crowding, regardless of the temperature level. Athanassiou et al. (2014) found that the population growth of psocids (Psocoptera) was mostly regulated with food availability and was eventually drastically reduced with the reduction of food.

Our study shows that the resistant population had clear advantages in progeny production over the numbers of the susceptible population. This is particularly important and denotes that the resistant population can have a considerable spread and colonization within the storage facility. Nonetheless, we are unaware if the differences recorded here are due to the occurrence of resistance or possible interactions of certain biological parameters with other biotic and abiotic factors, such as temperature or diet.

## Data Availability

The authors confirm that the data supporting the findings of this study are available within the article.
